# Cancer worry among *BRCA1/2* pathogenic variant carriers choosing surgery to prevent tubal/ovarian cancer: course over time and associated factors

**DOI:** 10.1007/s00520-021-06726-4

**Published:** 2022-01-08

**Authors:** Majke H. D. van Bommel, Miranda P. Steenbeek, Joanna IntHout, Rosella P. M. G. Hermens, Nicoline Hoogerbrugge, Marline G. Harmsen, Helena C. van Doorn, Marian J. E. Mourits, Marc van Beurden, Ronald P. Zweemer, Katja N. Gaarenstroom, Brigitte F. M. Slangen, Monique M. A. Brood-van Zanten, M. Caroline Vos, Jurgen M. Piek, Luc R. C. W. van Lonkhuijzen, Mirjam J. A. Apperloo, Sjors F. P. J. Coppus, Judith B. Prins, José A. E. Custers, Joanne A. de Hullu

**Affiliations:** 1 Department of Obstetrics and Gynaecology, Radboud Institute for Health Sciences, Radboud University Medical Center, PO Box 9101, 6500 HB Nijmegen, The Netherlands; 2 Department for Health Evidence, Radboud Institute for Health Sciences, Radboud University Medical Center, PO Box 9101, 6500 HB Nijmegen, The Netherlands; 3 Scientific Institute for Quality of Healthcare, Radboud Institute for Health Sciences, Radboud University Medical Center, PO Box 9101, 6500 HB Nijmegen, The Netherlands; 4 Department of Human Genetics, Radboud University Medical Center, PO Box 9101, 6500 HB Nijmegen, The Netherlands; 5 Department of Gynaecology, Erasmus MC Cancer Clinic, ‘s-Gravendijkwal 230, 3015 CE Rotterdam, The Netherlands; 6 Department of Gynaecologic Oncology, University Medical Center Groningen, University of Groningen, Hanzeplein 1, 9713 GZ Groningen, The Netherlands; 7 Center for Gynaecological Oncology Amsterdam (CGOA), Netherlands Cancer Institute/Antoni Van Leeuwenhoek Hospital, Plesmanlaan 121, 1066 CX Amsterdam, The Netherlands; 8 Department of Gynaecological Oncology, UMC Utrecht Cancer Center, Heidelberglaan 100, 3584 CX Utrecht, The Netherlands; 9 Department of Obstetrics and Gynaecology, Leiden University Medical Center, Albinusdreef 2, 2333 ZA Leiden, The Netherlands; 10 Department of Obstetrics and Gynaecology, Maastricht University Medical Center, GROW-School for Oncology and Developmental Biology, P. Debyelaan 25, 6229 HX Maastricht, The Netherlands; 11 Center for Gynaecological Oncology Amsterdam (CGOA), AmsterdamUMC, Meibergdreef 9, 1105 AZ Amsterdam, The Netherlands; 12 Gynaecologic Oncologic Center South Location Elisabeth-TweeSteden Hospital, Hilvarenbeekseweg 60, 5000 LC Tilburg, The Netherlands; 13 Gynaecologic Oncologic Center South Location Catharina Hospital, Department of Obstetrics and Gynaecology and Catharina Cancer Institute, Michelangelolaan 2, 5623 EJ Eindhoven, The Netherlands; 14 Department of Obstetrics and Gynaecology, Medical Center Leeuwarden, Henri Dunantweg 2, 8934 AD Leeuwarden, The Netherlands; 15 Department of Obstetrics and Gynaecology, Maxima Medical Center, De Run 4600, 5504 DB Veldhoven, The Netherlands; 16 Department of Medical Psychology, Radboud Institute F Or Health Sciences, Radboud University Medical Center, PO Box 9101, 6500 HB Nijmegen, The Netherlands

**Keywords:** *BRCA* gene, Cancer worry, Psychology, Ovarian cancer, Risk-reducing salpingo-oophorectomy, Salpingectomy

## Abstract

**Objective:**

High cancer risks, as applicable to *BRCA1* and *BRCA2* pathogenic variant (PV) carriers, can induce significant cancer concerns. We examined the degree of cancer worry and the course of this worry among *BRCA1/2*-PV carriers undergoing surgery to prevent ovarian cancer, and identified factors associated with high cancer worry.

**Methods:**

Cancer worry was evaluated as part of the multicentre, prospective TUBA-study (NCT02321228) in which *BRCA1/2*-PV carriers choose either novel risk-reducing salpingectomy with delayed oophorectomy or standard risk-reducing salpingo-oophorectomy. The Cancer Worry Scale was obtained before and 3 and 12 months after surgery. Cancer worry patterns were analysed using latent class growth analysis and associated factors were identified with regression analysis.

**Results:**

Of all 577 *BRCA1/2*-PV carriers, 320 (57%) had high (≥ 14) cancer worry pre-surgery, and 54% had lower worry 12 months post-surgery than pre-surgery. Based on patterns over time, *BRCA1/2*-PV carriers could be classified into three groups: persistently low cancer worry (56%), persistently high cancer worry (6%), and fluctuating, mostly declining, cancer worry (37%). Factors associated with persistently high cancer concerns were age below 35 (*BRCA1*) or 40 (*BRCA2*), unemployment, previous breast cancer, lower education and a more recent *BRCA1/2*-PV diagnosis.

**Conclusions:**

Some degree of cancer worry is considered normal, and most *BRCA1/2*-PV carriers have declining cancer worry after gynaecological risk-reducing surgery. However, a subset of these *BRCA1/2*-PV carriers has persisting major cancer concerns up to 1 year after surgery. They should be identified and potentially offered additional support.

**Clinical trial registration:**

The TUBA-study is registered at ClinicalTrials.gov since December 11th, 2014. Registration number: NCT02321228.

**Supplementary Information:**

The online version contains supplementary material available at 10.1007/s00520-021-06726-4.

## Introduction

Female carriers of a pathogenic variant (PV) in breast cancer (*BRCA*)1 or *BRCA2* gene are at high lifetime risk of developing breast (around 70%) and ovarian cancer (around 44% and 17% for *BRCA1/2*-PV carriers respectively) [1]. Ovarian cancer is typically diagnosed at an advanced-stage which contributes to the poor 5-year survival of about 45% [2, 3]. Currently, breast cancer risk management is based on annual screening for early detection or on primary prevention by risk-reducing mastectomy (RRM) [4]. For ovarian cancer, effective screening methods for early detection are not available [5–7]. Consequently, risk-reducing salpingo-oophorectomy (RRSO) is advised at the age of 35–40 (*BRCA1-*PV) or 40–45 years (*BRCA2*-PV carriers) [8].

Over the last two decades, the fallopian tube, instead of the ovary, has been identified as site of origin of most ovarian cancers [9, 10]. Since then, evidence for this has accumulated and a salpingectomy for the prevention of ovarian cancer among *BRCA1/2*-PV carriers was proposed. In the multicentre prospective TUBA-study (NCT02321228), a novel strategy of risk-reducing salpingectomy (RRS) with delayed oophorectomy (RRO) to delay premature menopause is investigated. *BRCA1/2*-PV carriers choose their preferred strategy: standard RRSO or the novel RRS with delayed RRO [11].

*BRCA1/2*-PV carriers may be prone to high levels of cancer worry. High cancer risk can induce cancer concerns. Undergoing surveillance and risk-reducing surgeries may increase cancer concerns. Furthermore, as a *BRCA1/2-*PV is transferred in an autosomal dominant manner, many *BRCA1/2*-PV carriers have experienced cancer-related morbidity and mortality in their families which can also influence cancer worry [12]. Additionally, the 50% risk to pass the *BRCA1/2-*PV to a child may impact cancer worry. Altogether, various factors and life stages play a role in cancer concerns that may affect *BRCA1/2*-PV carriers.

Some degree of cancer worry is considered normal and functional as it can keep persons aware of symptoms. However, a substantial proportion of cancer patients and cancer survivors was found to have high levels of cancer worry [13–17]. High cancer worry can cause significant emotional and social dysfunction which negatively affects quality of life [18]. Moreover, elevated levels of cancer worry can limit adherence to screening programs and may be a significant factor in decision-making about risk-reducing surgeries [19, 20]. Therefore, identifying women with high cancer worry is of great importance in order to offer accurate support. Thus far, data on cancer worry in *BRCA1/2*-PV carriers are very limited, especially regarding the course of cancer worry over time. Only one study, executed by Finch et al., investigated ovarian cancer-distress over time among *BRCA1/2*-PV carriers [21]. In our study, we aim to explore levels of cancer worry and the course of cancer worry in *BRCA1/2*-PV carriers undergoing surgery to prevent ovarian cancer. Secondary, we aim to assess predictors for high cancer worry.

## Methods

### Design and population

We evaluated cancer worry as part of the multicentre prospective preferential TUBA-study (NCT02321228). Details of the TUBA-study have been published previously [11]. Briefly, quality of life is investigated in *BRCA1/2*-PV carriers who choose their preferred surgery to reduce ovarian cancer risk: a standard RRSO or a novel strategy of RRS with delayed RRO. In the current study, all participants of the TUBA-study were included, being premenopausal *BRCA1/2*-PV carriers, aged 25 to 45 years who completed childbearing. Exclusion criteria were a history of ovarian cancer or treatment for any malignancy at enrolment. Inclusion was performed between January 2015 and November 2019 in thirteen Dutch hospitals. The TUBA-study is conducted in accordance with the principles of the Declaration of Helsinki and was approved by the Medical Ethics Committee of Arnhem-Nijmegen (registration number 2014–1269). Each participant signed informed consent.

### Outcome measures and data collection

In this study, we focused on cancer worry which was among the secondary outcomes of the TUBA-study. Data regarding cancer worry until 12 months post-surgery were evaluated. Cancer worry was assessed by the Dutch translation of the Cancer Worry Scale (CWS): a validated questionnaire to measure the worry about developing cancer (again) and its impact on daily functioning [22–24]. Eight items were scored on a four-point Likert-scale resulting in a score ranging from 8 to 32 points. A higher score represents more cancer worry. A score of ≥ 14 represents a high level of cancer worry [16].

All data in the TUBA-study, except the surgical and histopathological outcomes, were collected digitally with validated questionnaires and questions regarding baseline characteristics and perceived cancer risks. Perceived breast cancer risk and perceived ovarian cancer risk were scored on a scale from 0 (perceived risk of developing cancer 0%) to 100 (perceived risk of developing cancer 100%). In the questionnaires, we asked about current or previous severe anxiety, burn-out and depression and summarized these items as ‘emotional instability’ in this paper. The questionnaires were sent at baseline (either pre-RRSO or pre-RRS), 3 and 12 months post-surgery and then biennially.

### Data analysis

#### Analysis of cancer worry over time

Baseline data were reported with descriptive statistics. To calculate change of cancer worry over time, an absolute change score (delta) was calculated for the intervals between pre- and 3 months post-surgery and between pre- and 12 months post-surgery. In order to analyse trajectories of cancer worry, we used two methods: one based on a predefined cutoff score and one data-driven approach. In the first method, participants who completed the questionnaire at all three time points were classified into one of the following groups based on the cutoff score: (1) persistently low: cancer worry score below 14 at all three time points, (2) fluctuating: at least one cancer worry score equal to or above and one below 14 at any of the three time points, or (3) persistently high: cancer worry score of 14 or higher at all three time points.

In the second, data-driven, analysis, we conducted latent class growth analysis (LCGA) to classify women into classes with similar patterns of cancer worry over time [25]. All women who were at least 1 year postoperative irrespective of completion of the questionnaires were included. Full information maximum likelihood estimation for handling missing data was applied. A single-class growth curve model, as well as a two-, three-, four- and five- class model, was specified. To determine the most appropriate number of classes for our data, models were compared on model parsimony, fit indices and clinical interpretability. The best model fit indices ideally correspond to significant *p-*values for the bootstrap likelihood ratio and the Vuong-Lo-Mendel Ruben likelihood ratio test, the smallest Bayesian information criterion (BIC) and higher entropy and posterior probabilities of group membership. A minimum number of participants (≥ 5% of total sample) in a class were required for clinical interpretability. We used Mplus version 8.3 to conduct LCGA.

#### Analysis of associated variables

A multivariable linear regression analysis was performed with all variables that theoretically might relate to preoperative cancer worry (dependent variable). The independent variables included age, *BRCA1/2*-PV type, years since *BRCA1/2*-PV diagnosis, educational level, offspring, employment status, relationship status, previous RRM, personal history of breast cancer or other cancers, history of or current emotional instability, antidepressants use, familial history of breast and/or ovarian cancer, chosen risk-reducing surgery (RRSO or RRS), perceived ovarian cancer risk and perceived breast cancer risk. This multivariable analysis was performed using the backward-stepwise method (*p*-in 0.05 and *p*-out 0.10) and two-sided *p*-values below 0.05 were considered statistically significant.

Variables associated with cancer worry trajectories as identified with predefined cutoff scores were compared between the three subgroups using Chi-square or Kruskal–Wallis tests. To analyse variables associated with the classes as identified in the LCGA, we conducted a multinomial logistic regression analysis per variable. Here, class of cancer worry pattern was the dependent variable, and the independent variables were equal to those in the linear regression as mentioned in the previous paragraph. All analyses, except the LCGA, were performed in SPSS version 25 [26].

## Results

A total of 577 women participated in the TUBA-study. The questionnaire pre-surgery was completed by 96.9%, 3 months post-surgery by 96.9% and 12 months post-surgery by 94.5% of the participants that had passed the respective time points (Online Resource 1). Missing data was considered to be at random since baseline characteristics were similar between women who did and did not complete the baseline questionnaire and because the main reason for missing at 3 and 12 months was waiting for surgery or follow-up. All women had a mean age of 37.2 years at inclusion, and 51% carried a *BRCA1*-PV. Educational level was high in 51%. Breast cancer was previously diagnosed in 14%, and 38% had undergone RRM. Current or previous psychological problems, e.g. severe anxiety, burn-out and/or depression as reported by the women themselves (summarized as emotional instability) was present in 17% (Table 1).

**Table 1:** Baseline characteristics

	*BRCA1/2*-PV carriers (*n* = 577)	
	Mean/*N*	SD/%
Age, years	37.2	3.5
Pathogenic variant		
* BRCA1*	297	51.5%
* BRCA2*	280	48.5%
Years since *BRCA1/2*-PV diagnosis	5.0	4.6
Chosen risk-reducing surgery		
RRSO	164	28.4%
RRS with delayed RRO	413	71.6%
Educational level		
Low	64	11.1%
Intermediate	205	35.5%
High	296	51.3%
Unknown	12	2.1%
Employment status		
Employed	466	80.8%
Unemployed	92	15.9%
Unknown	17	2.9%
Relationship status		
Married/relationship	511	88.6%
Single/divorced/widowed	54	9.4%
Unknown	12	2.1%
Offspring		
Yes	497	86.1%
No	65	11.3%
Unknown	15	2.6%
History of cancer		
Breast	81	14.3%
Other^a^	5	0.9%
Risk-reducing mastectomy		
Yes	223	38.6%
No	342	59.3%
Unknown	12	2.1%
(History of) emotional instability^b^		
Yes	97	16.8%
No	468	81.1%
Unknown	12	2.1%
Medication use		
Antidepressants	32	5.5%
Antipsychotics	1	0.2%
Benzodiazepines	10	1.7%
First degree family history		
Breast cancer	266	6.1%
Ovarian cancer	77	13.3%

^a^Other cancers included non-melanoma skin cancer (3 women), cervical cancer

(1 woman) and an appendicular neoplasm (1 woman)

^b^Emotional instability is defined as self-reported current or previous severe anxiety, burn-out and/or depression

### Cancer worry levels

**Fig. 1:**
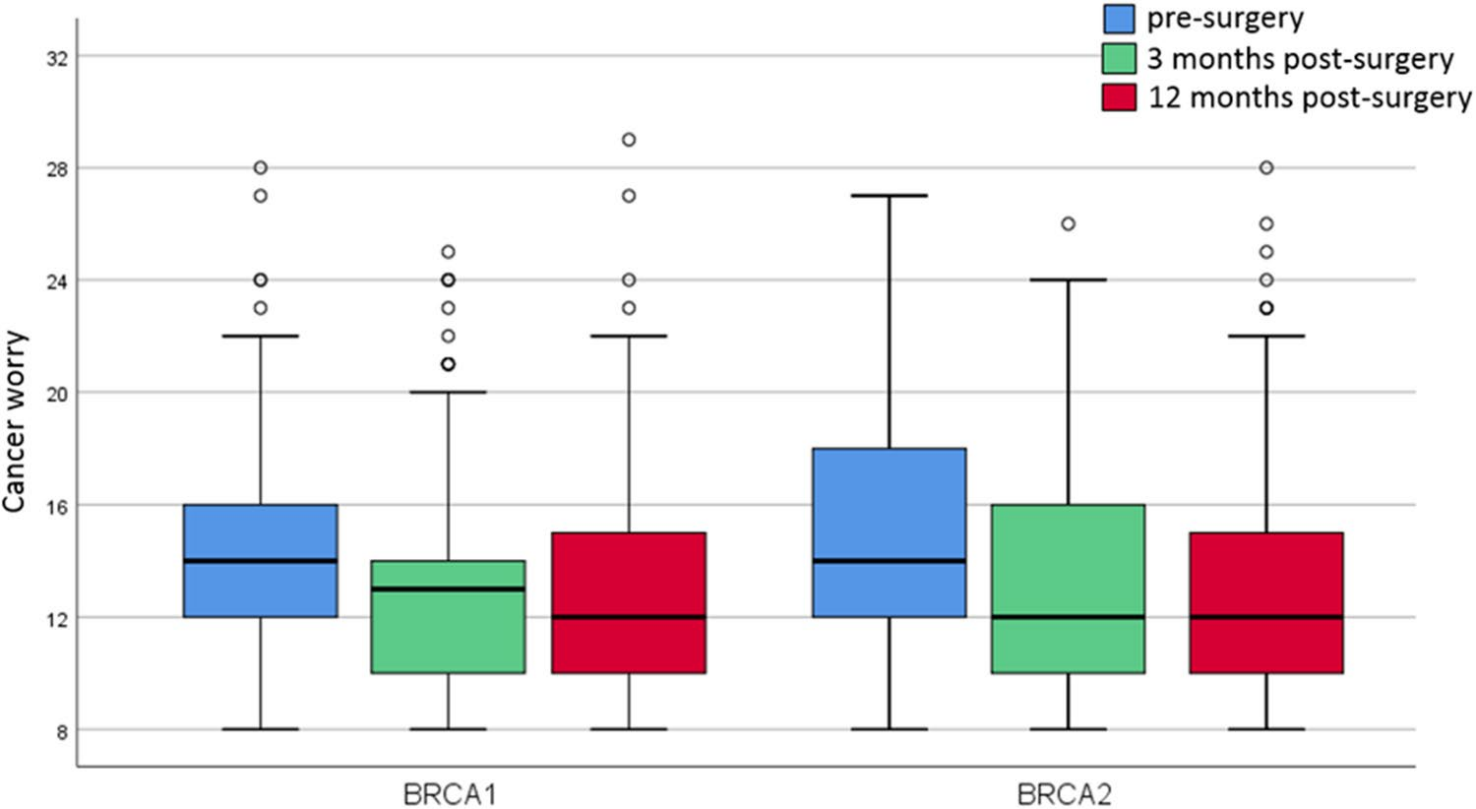
Cancer worry pre- and 3 and 12 months post-surgery. Figure was created in SPSS

Median cancer worry level before surgery (RRSO or RRS) was 14 (interquartile range (IQR) 12; 16) for *BRCA1*-PV and 14 (IQR 12; 18) for *BRCA2*-PV carriers. High levels of cancer worry (≥ 14) were identified in 320 women pre-surgery: 57% of the *BRCA1*-PV and 58% of the *BRCA2*-PV carriers. Three months post-surgery, median cancer worry declined to 13 (IQR 10; 14) for *BRCA1*-PV and to 12 (IQR 10; 16) for *BRCA2*-PV carriers. At that moment, 37% of *BRCA1*-PV and 38% of the *BRCA2*-PV carriers had high cancer worry. Twelve months post-surgery, median cancer worry was 12 (IQR 10; 15) for both *BRCA1*-PV and *BRCA2*-PV carriers. Then, high cancer worry was present in 38% and 36% of the *BRCA1/2*-PV carriers respectively. Overall, compared to pre-surgery, 3 months post-surgery cancer worry score was lower in 58% (median delta − 1.5 points, IQR − 3; 0) and 12 months post-surgery in 54% (median delta − 2 points, IQR − 4; 0). No notable differences were found between women choosing RRSO or RRS with delayed RRO. Between women with or without previous RRM, pre- and 12 months post-surgery median cancer worry scores were similar, while 3 months post-surgery women with RRM scored median 11 (IQR 10; 14) and women without RRM median 13 (IQR 11; 15). Figure 1 visualizes cancer worry scores at the three time points.

### Cancer worry trajectories

To distinct groups based on the predefined cutoff score, complete longitudinal data was available for 488 women. Of these, 173 (36%) had persistently low levels of cancer worry (< 14 at all three time points). A total of 209 (43%) had fluctuating levels of cancer worry of which 80% had high cancer worry preoperative that declined afterwards. Persistently, high levels of cancer worry (≥ 14 at all three time points) were found in 106 (22%) *BRCA1/2*-PV carriers (55 *BRCA1*, 51 *BRCA2*).

Using LCGA, we included 525 women. A three-class model was considered most appropriate because of its fit indices and clinical interpretability (Online Resource 2). In this model, the classes had various baseline levels of cancer worry (intercepts) and differed in cancer worry scores over time (slopes). The first class consisted of 296 (56%) women who had pre-surgical low cancer worry (intercept 12.1, 95%CI 11.6; 12.6) with significantly declining cancer worry over time (slope − 0.9 per time point, 95%CI − 1.0; − 0.7). This class was defined as ‘low declining’. The second class, *n* = 33 (6%), was defined as ‘high stable’, as women had high baseline cancer worry (intercept 21.6, 95%CI 20.0; 23.1) that remained high over time (non-significant slope − 0.0, 95%CI − 0.7; 0.7). The third class, defined as ‘high declining’, entailed 196 (37%) women with high baseline cancer worry that decreased significantly over time (intercept 16.2, 95%CI 15.4; 17.0 and slope − 0.9, 95%CI − 1.2; − 0.6).

### Factors associated with preoperative cancer worry

Linear regression analysis resulted in eight variables significantly associated with higher pre-surgical cancer worry. These variables explained together 8.1% of the total variation in cancer worry (Table 2). *BRCA2*-PV carriers had a 0.8 points higher cancer worry than *BRCA1*-PV carriers. Increasing time since *BRCA1/2-*PV diagnosis and being employed were significantly associated with lower cancer worry. Previous breast cancer, emotional instability or RRM were significantly associated with higher cancer worry.

**Table 2:** Variables associated with high preoperative cancer worry

		Multivariable linear regression		
		*β*	95%CI	*p*-value
*BRCA1/2*-PV type	1	0		
	2	0.842	0.138; 1.545	0.019
Years since *BRCA1/2-*PV diagnosis		− 0.080	− 0.150; − 0.009	0.028
History of breast cancer	No	0		
	Yes	1.301	0.402; 2.201	0.005
Emotional instability	No	0		
	Yes	1.231	0.419; 2.044	0.003
Previous risk-reducing mastectomy	No	0		
	Yes	0.961	0.120; 1.803	0.025
Working	No	0		
	Yes	− 0.980	− 1.799; − 0.161	0.019
Breast cancer risk perception (scale 0–100)		0.016	0.003; 0.028	0.017
Ovarian cancer risk perception (scale 0–100)		0.033	0.017; 0.049	0.000

Adjusted *R*^2^ = 0.081

*β*, unstandardized beta, represents the slope of the line between the predictor variable and the dependent variable (cancer worry); *CI*, confidence interval; *BRCA1/2*-PV, *BRCA1/2* pathogenic variant

### Factors associated with cancer worry trajectories

Compared to women in the fluctuating or persistently low cancer worry group, women in the persistently high cancer worry group have had breast cancer more often (9% vs 11% vs 31% respectively, *p* < 0.001). Emotional instability was more frequently reported in women in the fluctuating or persistently high cancer worry group compared to women in the persistently low cancer worry group (20% vs 23% vs 11%, *p* = 0.021). Other variables associated with cancer worry did not significantly differ between the groups (Online Resource 3).

Results of the multinomial logistic regression analysis comparing baseline characteristics between the three classes with different patterns of cancer worry over time (LCGA) are shown in Table 3 and Online Resource 4. Seven variables were significantly associated with the classes (Table 3). Compared to women without breast cancer, women with previous breast cancer were significantly more likely to belong to the high stable class (odds ratio (OR) 4.2) or the high declining class (OR 2.8). Women with more time since *BRCA1/2*-PV diagnosis were less likely to have high stable (OR 0.9) or high declining cancer worry (OR 1.0). Women in the high stable class were significantly less likely to be within the guideline age range for RRSO (*BRCA1*-PV: 35–40 years, *BRCA2-*PV: 40–45 years) (OR 0.4), be employed (OR 0.4) and be higher educated (OR 0.6). Women with emotional instability or higher ovarian cancer risk perception were more likely to belong to the high declining class (OR 2.1 and 1.0 respectively).

**Table 3:** Characteristics associated with membership in a class with similar patterns of cancer worry over time; low declining versus high stable and high declining class

	Class	Multinomial logistic regression		
		OR	95%CI	*p*-value
Within guideline age	Low declining	1		
	High stable	0.390	0.185; 0.822	0.013
	High declining	1.174	0.810; 1.703	0.397
Employed	Low declining	1		
	High stable	0.401	0.178; 0.907	0.028
	High declining	1.235	0.736; 2.072	0.425
History of breast cancer	Low declining	1		
	High stable	4.226	1.766; 10.115	0.001
	High declining	2.769	1.618; 4.739	< 0.001
Emotional instability	Low declining	1		
	High stable	1.609	0.621; 4.170	0.328
	High declining	2.138	1.327; 3.447	0.002
High educational level	Low declining	1		
	High stable	0.576	0.350; 0.947	0.030
	High declining	0.981	0.751; 1.282	0.888
Ovarian cancer risk perception (per point of increased perceived risk)	Low declining	1		
	High stable	1.016	1.000; 1.033	0.054
	High declining	1.010	1.001; 1.018	0.024
Years since *BRCA1/2*-PV diagnosis	Low declining	1		
	High stable	0.873	0.790; 0.965	0.008
	High declining	0.955	0.917; 0.994	0.024

*OR*, Odds ratio; *CI*, confidence interval

## Discussion

In this study, we analysed cancer worry and the course of cancer worry over time among *BRCA1/2*-PV carriers up to 12 months after surgery to prevent ovarian cancer. We observed high cancer worry prior to risk-reducing gynaecological surgery in 57% of all *BRCA1/2*-PV carriers, without differences between women who chose RRS or RRSO. Cancer concerns declined after surgery in most women, suggesting that most women find a way to deal with their cancer concerns. However, a substantial subset (6% and 22%) had persistent major cancer concerns up to a year after preventive surgery. Women with persistently high cancer worry scores were more likely to be below age 35 (*BRCA1*) or 40 (*BRCA2*) years, be unemployed, have had breast cancer, be lower educated and have shorter time between *BRCA1/2*-PV diagnosis and surgery. In this particular group, it appeared that surgery did not reduce cancer worry.

This is the largest series that prospectively measures cancer worry in *BRCA1/2*-PV carriers over time. Our findings are in line with the only other study that investigated distress over time in *BRCA1/2*-PV carriers, as they found declining scores [21]. Moreover, other studies seem to support our findings too as they found higher cancer worry in women at high risk for breast and ovarian cancer who had not (yet) undergone RRSO compared to women who had [27–30]. We should take into account that the concern levels that we measured may be higher than the concerns in daily life, as our measurements were in the period around surgery which may be a period with increased cancer concerns in general. We found higher percentages of women with high cancer worry than Finch et al. [21] did which may have several explanations. First, we assessed cancer worry instead of specific ovarian cancer–related distress. A part of the cancer worry may be explained by the high risk of developing breast cancer that remains after gynaecological surgery. Second, since our participants choose their risk-reducing strategy themselves, they might feel ‘responsible’ for their choice, which may have heightened their cancer concerns. Though, cancer worry did not differ between women choosing RRSO or RRS. Third, age at surgery could explain some of higher worries that we found as our participants were approximately 10 years younger. A younger age was previously proven to be associated with higher worry [18, 31, 32]. However, ‘age’ itself was not among our identified predictors for high cancer worry, possibly due to insufficient discriminating power because we included women within a limited age range. Further, the proportion of women with emotional instability in our study is an unlikely explanation as the prevalence of emotional instabilities we found (17%) was quite similar to the prevalence of anxiety (19.6%) or mood disorders (20.2%) in the general Dutch population [33].

Interestingly, in our study, the proportion of women with high cancer worry (57%) is almost equal to a study that evaluated fear of cancer recurrence with the Cancer Worry Scale among adolescent and young adult (AYA) cancer patients; 62% had high levels of fear [34]. Moreover, absolute cancer worry scores were almost similar between AYA cancer patients and our *BRCA1/2*-PV carriers opting for preventive surgery. Also, in 70% of early-stage breast cancer survivors aged 18 to 45 years clinical levels of fear of cancer recurrence were observed [35]. This indicates that, in young adults, having a high risk of getting cancer or an actual cancer diagnosis, has a similar influence on cancer worry. In other high-risk patients, for example with familial adenomatous polyposis, health-related quality of life was comparable to that of the general population [36]. Contrastingly, 28% of the patients counselled for Lynch syndrome developed a clinically significant level of cancer-related distress [37]. These findings demonstrate that cancer worry is a frequent issue, not only for young cancer patients, but also for people at high risk of various cancers.

Cancer worry was related to time since *BRCA1/2*-PV diagnosis and being before or within the guideline age for RRSO (*BRCA1*: 35–40 years and *BRCA2:* 40–45 years). Thus, with respect to cancer worry, it seems beneficial to stretch the interval between *BRCA1/2*-PV diagnosis and risk-reducing surgery. To stretch this interval, two aspects may be important: first, diagnosing *BRCA1/2*-PV carriership at a relatively young age and second, being conservative with surgery at young age. Regarding the first aspect, potentially the Tumor-First workflow will contribute to more frequent and earlier knowledge of a hereditary *BRCA1/2*-PV within a family [38]. In Tumor-First, universal *BRCA1/2*-PV tumour testing in all new epithelial ovarian cancer patients is performed (instead of only testing women who request referral). Therefore, increasing numbers of *BRCA1/2*-PVs are detected, offering opportunities for testing and prevention amongst family members. Regarding the second aspect, it should be realized by both the doctor and the patient that surgery is not the best treatment for anxiety or worry. This applies especially to young *BRCA1/2*-PV carriers since cancer risks are still low at young age, cancer worry decreases with an increasing time since *BRCA* diagnosis irrespective of surgery, and, in general, the younger age at sterilizing surgery the higher the risk of regret of this surgery [39].

For clinical practice, our results should be included in counselling *BRCA1/*2-PV carriers about expectations of cancer concerns over time. We should aim to identify women at risk for high worry and offer them easily accessible psychological support. Blended cognitive behaviour therapy was proved efficacious for high fear of cancer recurrence in survivors of various types of cancer [40–42]. Therefore, this type of treatment could also be beneficial for women with persistently high cancer worry. In future research, it would be worth investigating whether blended cognitive behaviour therapy could be extrapolated to women at high risk for cancer.

Main strengths of this study are the prospective multicentre design with many participants and all-time extremely high response rates. Another strength is the use of both a validated cutoff (based on clinical knowledge) and a data-driven (statistical) approach to define cancer worry patterns. Both approaches identified three distinct groups in cancer worry course which improves validity. But, we should take into account that only linear trajectories were assessed. Also, we should be aware of the nonrandomized design. Moreover, RRS is currently strictly recommended within the context of a clinical trial and only performed in participating hospitals, whereas RRSO can be performed in every hospital in the Netherlands. Thus, probably not all women preferring RRSO were referred to a participating hospital while almost all women choosing RRS were referred. Women that requested referral to a participating hospital may be different from non-referred women.

In conclusion, some degree of cancer worry is considered normal, and most cancer concerns decline after risk-reducing surgery. However, major cancer concerns remain present in a smaller, but substantial proportion of the *BRCA1/2*-PV carriers who undergo risk-reducing gynaecological surgery. Women at risk for these persisting major concerns are aged below 35 (*BRCA1*) or 40 (*BRCA2*) years*,* unemployed, lower educated, have a history of breast cancer or a more recent *BRCA1/2-*PV diagnosis. Identifying these women is important as they could potentially benefit from psychological support. We recommend including this knowledge about cancer worry in counselling about expectations of cancer concerns over time and timing of surgery. Additionally, we do not recommend performing surgery on women before the guideline age for RRSO when their motive for surgery is based on fear.

## Supplementary Information

Below is the link to the electronic supplementary material.

Supplementary file1 (PDF 136 KB)

Supplementary file2 (PDF 111 KB)

Supplementary file3 (PDF 115 KB)

Supplementary file4 (PDF 122 KB)

## Data Availability

Data used for this analysis are available upon reasonable request to the corresponding author.

## References

[1] Kuchenbaecker KB, Hopper JL, Barnes DR, Phillips KA, Mooij TM, Roos-Blom MJ (2017). Risks of breast, ovarian, and contralateral breast cancer for BRCA1 and BRCA2 mutation carriers. JAMA.

[2] Siegel RL, Miller KD, Jemal A (2019). Cancer statistics, 2019. CA Cancer J Clin.

[3] Lheureux S, Gourley C, Vergote I, Oza AM (2019). Epithelial ovarian cancer. Lancet.

[4] IntegraalKankercentrumNederland. Richtlijn Borstkanker (English: Guideline Breastcancer). Published 01–03–2017. Accessed 15 Apr 2021

[5] Henderson JT, Webber EM, Sawaya GF (2018). Screening for ovarian cancer: updated evidence report and systematic review for the US preventive services task force. JAMA.

[6] Oei AL, Massuger LF, Bulten J, Ligtenberg MJ, Hoogerbrugge N, de Hullu JA (2006). Surveillance of women at high risk for hereditary ovarian cancer is inefficient. Br J Cancer.

[7] van der Velde NM, Mourits MJ, Arts HJ, de Vries J, Leegte BK, Dijkhuis G (2009). Time to stop ovarian cancer screening in BRCA1/2 mutation carriers?. Int J Cancer.

[8] IntegraalKankercentrumNederland. Richtlijn Erfelijk en Familiair Ovariumcarcinoom (English: Guideline Hereditary and Familial Ovarian Carcinoma). Published 06–15–2015. Accessed 6 Oct

[9] Piek JM, van Diest PJ, Zweemer RP, Jansen JW, Poort-Keesom RJ, Menko FH (2001). Dysplastic changes in prophylactically removed fallopian tubes of women predisposed to developing ovarian cancer. J Pathol.

[10] Labidi-Galy SI, Papp E, Hallberg D, Niknafs N, Adleff V, Noe M (2017). High grade serous ovarian carcinomas originate in the fallopian tube. Nat Commun.

[11] Harmsen MG, Arts-de Jong M, Hoogerbrugge N, Maas AH, Prins JB, Bulten J (2015). Early salpingectomy (TUbectomy) with delayed oophorectomy to improve quality of life as alternative for risk-reducing salpingo-oophorectomy in BRCA1/2 mutation carriers (TUBA study): a prospective non-randomised multicentre study. BMC Cancer.

[12] Rees G, Fry A, Cull A (2001). A family history of breast cancer: women’s experiences from a theoretical perspective. Soc Sci Med.

[13] Mirosevic S, Thewes B, van Herpen C, Kaanders J, Merkx T, Humphris G (2019). Prevalence and clinical and psychological correlates of high fear of cancer recurrence in patients newly diagnosed with head and neck cancer. Head Neck.

[14] Custers JAE, Gielissen MFM, Janssen SHV, de Wilt JHW, Prins JB (2016). Fear of cancer recurrence in colorectal cancer survivors. Support Care Cancer.

[15] van de Wal M, van Oort I, Schouten J, Thewes B, Gielissen M, Prins J (2016). Fear of cancer recurrence in prostate cancer survivors. Acta Oncol.

[16] Custers JA, van den Berg SW, van Laarhoven HW, Bleiker EM, Gielissen MF, Prins JB (2014). The Cancer Worry Scale: detecting fear of recurrence in breast cancer survivors. Cancer Nurs.

[17] Gonçalves V, Jayson G, Tarrier N (2008). A longitudinal investigation of psychological morbidity in patients with ovarian cancer. Br J Cancer.

[18] Sharpe L, Turner J, Fardell JE, Thewes B, Smith AB, Gilchrist J (2019). Psychological intervention (ConquerFear) for treating fear of cancer recurrence: mediators and moderators of treatment efficacy. J Cancer Surviv.

[19] Andersen MR, Smith R, Meischke H, Bowen D, Urban N (2003). Breast cancer worry and mammography use by women with and without a family history in a population-based sample. Cancer Epidemiol Biomarkers Prev.

[20] Hurley KE, Miller SM, Costalas JW, Gillespie D, Daly MB (2001). Anxiety/uncertainty reduction as a motivation for interest in prophylactic oophorectomy in women with a family history of ovarian cancer. J Womens Health Gend Based Med.

[21] Finch A, Metcalfe KA, Chiang J, Elit L, McLaughlin J, Springate C (2013). The impact of prophylactic salpingo-oophorectomy on quality of life and psychological distress in women with a BRCA mutation. Psychooncology.

[22] Lerman C, Daly M, Masny A, Balshem A (1994). Attitudes about genetic testing for breast-ovarian cancer susceptibility. J Clin Oncol.

[23] Watson M, Duvivier V, Wade Walsh M, Ashley S, Davidson J, Papaikonomou M (1998). Family history of breast cancer: what do women understand and recall about their genetic risk?. J Med Genet.

[24] Douma KF, Aaronson NK, Vasen HF, Gerritsma MA, Gundy CM, Janssen EP (2010). Psychological distress and use of psychosocial support in familial adenomatous polyposis. Psychooncology.

[25] Jung T, Wickrama KAS (2008). An introduction to latent class growth analysis and growth mixture modeling. Soc Pers Psychol Compass.

[26] IBM Corp. Released 2017. IBM SPSS Statistics for Windows VA, NY: IBM Corp

[27] Powell CB, Alabaster A, Le A, Stoller N, Armstrong MA, Raine-Bennett T (2020). Sexual function, menopausal symptoms, depression and cancer worry in women with BRCA mutations. Psychooncology.

[28] Metcalfe KA, Price MA, Mansfield C, Hallett DC, Lindeman GJ, Fairchild A (2020). Predictors of long-term cancer-related distress among female BRCA1 and BRCA2 mutation carriers without a cancer diagnosis: an international analysis. Br J Cancer.

[29] Shigehiro M, Kita M, Takeuchi S, Ashihara Y, Arai M, Okamura H (2016). Study on the psychosocial aspects of risk-reducing salpingo-oophorectomy (RRSO) in BRCA1/2 mutation carriers in Japan: a preliminary report. Jpn J Clin Oncol.

[30] Michelsen TM, Dørum A, Dahl AA (2009). A controlled study of mental distress and somatic complaints after risk-reducing salpingo-oophorectomy in women at risk for hereditary breast ovarian cancer. Gynecol Oncol.

[31] Lebel S, Beattie S, Arès I, Bielajew C (2013). Young and worried: age and fear of recurrence in breast cancer survivors. Health Psychol.

[32] Linden W, Vodermaier A, Mackenzie R, Greig D (2012). Anxiety and depression after cancer diagnosis: prevalence rates by cancer type, gender, and age. J Affect Disord.

[33] de Graaf R, Ten Have M, van Gool C, van Dorsselaer S (2012). Prevalence of mental disorders, and trends from 1996 to 2009. Results from NEMESIS-2. Tijdschr Psychiatr.

[34] Thewes B, Kaal SEJ, Custers JAE, Manten-Horst E, Jansen R, Servaes P (2018). Prevalence and correlates of high fear of cancer recurrence in late adolescents and young adults consulting a specialist adolescent and young adult (AYA) cancer service. Support Care Cancer.

[35] Thewes B, Butow P, Bell ML, Beith J, Stuart-Harris R, Grossi M (2012). Fear of cancer recurrence in young women with a history of early-stage breast cancer: a cross-sectional study of prevalence and association with health behaviours. Support Care Cancer.

[36] Douma KF, Bleiker EM, Vasen HF, Gundy CM, Aaronson NK (2011). Quality of life and consequences for daily life of familial adenomatous polyposis (FAP) family members. Colorectal Dis.

[37] Keller M, Jost R, Kadmon M, Wüllenweber HP, Haunstetter CM, Willeke F (2004). Acceptance of and attitude toward genetic testing for hereditary nonpolyposis colorectal cancer: a comparison of participants and nonparticipants in genetic counseling. Dis Colon Rectum.

[38] Vos JR, Fakkert IE, de Hullu JA, van Altena AM, Sie AS, Ouchene H (2020). Universal tumor DNA BRCA1/2 testing of ovarian cancer: prescreening PARPi treatment and genetic predisposition. J Natl Cancer Inst.

[39] Curtis KM, Mohllajee AP, Peterson HB (2006). Regret following female sterilization at a young age: a systematic review. Contraception.

[40] Burm R, Thewes B, Rodwell L, Kievit W, Speckens A, van de Wal M (2019). Long-term efficacy and cost-effectiveness of blended cognitive behavior therapy for high fear of recurrence in breast, prostate and colorectal cancer survivors: follow-up of the SWORD randomized controlled trial. BMC Cancer.

[41] van de Wal M, Thewes B, Gielissen M, Speckens A, Prins J (2017). Efficacy of blended cognitive behavior therapy for high fear of recurrence in breast, prostate, and colorectal cancer survivors: the SWORD study, a randomized controlled trial. J Clin Oncol.

[42] Tauber NM, O'Toole MS, Dinkel A, Galica J, Humphris G, Lebel S (2019). Effect of psychological intervention on fear of cancer recurrence: a systematic review and meta-analysis. J Clin Oncol.

