# Supplementary material for: Cancer worry among BRCA1/2 pathogenic variant carriers choosing surgery to prevent tubal/ovarian cancer: course over time and associated factors
Source: Support Care Cancer. 2022 Jan 8;30(4):3409–18. doi: 10.1007/s00520-021-06726-4 (PMC8857097; doi:10.1007/s00520-021-06726-4)

Cancer Worry among *BRCA1/2* pathogenic variant carriers choosing surgery to prevent tubal/ovarian cancer: course over time and associated factors

Majke H.D. van Bommel[1], Miranda P. Steenbeek[1], Joanna IntHout[2], Rosella P.M.G. Hermens[3], Nicoline Hoogerbrugge[4], Marline G. Harmsen[1], Helena C. van Doorn[5], Marian J.E. Mourits[6], Marc van Beurden[7], Ronald P. Zweemer[8], Katja N. Gaarenstroom[9], Brigitte F.M. Slangen[10], Monique M.A. Brood-van Zanten[7,11], M. Caroline Vos[12], Jurgen M. Piek[13], Luc R.C.W. van Lonkhuijzen[11], Mirjam J.A. Apperloo[14], Sjors F.P.J. Coppus[15], Judith B. Prins[16], José A.E. Custers[16], Joanne A. de Hullu[1]

Corresponding author

Name: Majke van Bommel

Affiliation: Radboud university medical center, Radboud Institute for Health Sciences, Department of Obstetrics and Gynaecology, Nijmegen, The Netherlands.

E-mail address: majke.vanbommel@radboudumc.nl

Online Resource 1. *Flow diagram of participating BRCA1/2-PV carriers (CONSORT)*

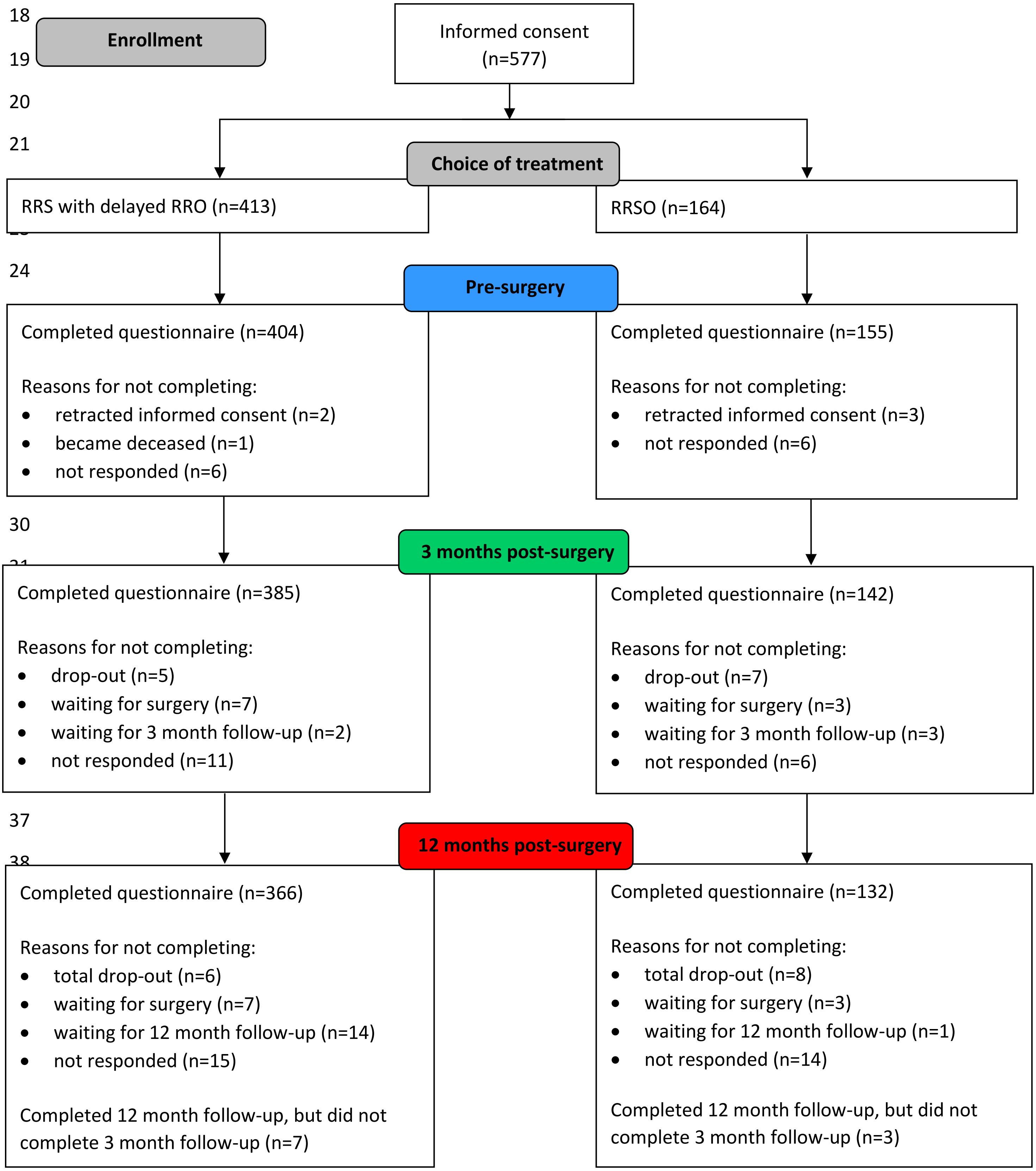

Supplement: Supplementary file 1 — Supplementary file1 (PDF 136 KB) [file 520_2021_6726_MOESM1_ESM.pdf]
