# Supplementary material for: Cancer worry among BRCA1/2 pathogenic variant carriers choosing surgery to prevent tubal/ovarian cancer: course over time and associated factors
Source: Support Care Cancer. 2022 Jan 8;30(4):3409–18. doi: 10.1007/s00520-021-06726-4 (PMC8857097; doi:10.1007/s00520-021-06726-4)
Supplement: Supplementary file 2 — Supplementary file2 (PDF 111 KB) [file 520_2021_6726_MOESM2_ESM.pdf]

Cancer Worry among *BRCA1/2* pathogenic variant carriers choosing surgery to prevent tubal/ovarian cancer: course over time and associated factors

Majke H.D. van Bommel[1], Miranda P. Steenbeek[1], Joanna IntHout[2], Rosella P.M.G. Hermens[3], Nicoline Hoogerbrugge[4], Marline G. Harmsen[1], Helena C. van Doorn[5], Marian J.E. Mourits[6], Marc van Beurden[7], Ronald P. Zweemer[8], Katja N. Gaarenstroom[9], Brigitte F.M. Slangen[10], Monique M.A. Brood-van Zanten[7,11], M. Caroline Vos[12], Jurgen M. Piek[13], Luc R.C.W. van Lonkhuijzen[11], Mirjam J.A. Apperloo[14], Sjors F.P.J. Coppus[15], Judith B. Prins[16], José A.E. Custers[16], Joanne A. de Hullu[1]

Corresponding author

Name: Majke van Bommel

Affiliation: Radboud university medical center, Radboud Institute for Health Sciences, Department of Obstetrics and Gynaecology, Nijmegen, The Netherlands.

E-mail address: majke.vanbommel@radboudumc.nl

Online Resource 2. *Parameters of the LCGA-models with different number of classes*

No. of classes	BIC	LMR-LRT	BLRT	Entropy	n	Posterior Probabi-lities	Intercept (95% CI)	Slope linear (95% CI) (per time point)
1	7772.891						14.24 (13.91; 14.56)***	-0.84 (-0.99; 0.69)***
2	7934.789	.0005	.0000	.873	87 (16.6%)	.952	19.33 (17.62; 21.04)***	-0.74 (-1.3; 0.17)**
					438 (83.4%)	.967	13.12 (12.75; 13.5)***	-0.89 (-1.04; 0.74)***
3	7768.655	.0022	.0000	.804	296 (56.4%)	.918	12.08 (11.61; 12.56)***	-0.87 (-1.02; 0.71)***
					33 (6.3%)	.959	21.56 (19.99; 23.13)***	-0.01 (-0.69; 0.66)
					196 (37.3%)	.879	16.2 (15.41; 16.98)***	-0.91 (-1.23; 0.59)***
4	7711.554	.0052	.0000	.790	225 (42.9%)	.862	14.76 (14.32; 15.21)***	-0.83 (-1.12; 0.54)***
					24 (4.6%)	.920	22.44 (20.56; 24.32)***	0.09 (-0.84; 1.02)
					60 (11.4%)	.879	18.89 (17.76; 20.03)***	-1.18 (-2.09; 0.27)*
					216 (41.1%)	.899	11.49 (11.13; 11.86)***	-0.86 (-1.03; 0.7)***
5	7703.974	.1633	.0000	.789	27 (5.1%)	.930	22.17 (20.61; 23.73)***	0.16 (-0.66; 0.99)
					31 (5.9%)	.830	20.45 (19.6; 21.31)***	-2.82 (-4; 1.64)***
					195 (37.1%)	.900	11.4 (11.03; 11.77)***	-0.86 (-1.04; 0.68)***
					48 (9.1%)	.797	15.91 (14.14; 17.67)***	0.87 (0.21; 1.52)**
					224 (42.7%)	.839	14.62 (14.18; 15.07)***	-0.97 (-1.3; 0.64)***

BIC, Bayesian Information Criterion; LMR-LRT, Vuong-Lo-Mendell Rubin Likelihood Ratio Test; BLRT, Bootstrap Likelihood Ratio Test; CI, confidence interval

*p<0.05. **p<0.01. ***p<0.001

The three-class model was considered most appropriate because of its fit indices and clinical interpretability. In the three-class model, compared with the two-class model, the BIC was better, but the entropy was lower. The posterior probabilities were similar for the two and three-class model. Compared with the four-class mode,. in the three-class model, the BIC was somewhat higher, whereas other fit indices were similar. In the four-class model, however, a class was identified including only 24 (<5%) women, limiting clinical interpretability.
