# Supplementary material for: Cancer worry among BRCA1/2 pathogenic variant carriers choosing surgery to prevent tubal/ovarian cancer: course over time and associated factors
Source: Support Care Cancer. 2022 Jan 8;30(4):3409–18. doi: 10.1007/s00520-021-06726-4 (PMC8857097; doi:10.1007/s00520-021-06726-4)
Supplement: Supplementary file 3 — Supplementary file3 (PDF 115 KB) [file 520_2021_6726_MOESM3_ESM.pdf]

Cancer Worry among *BRCA1/2* pathogenic variant carriers choosing surgery to prevent tubal/ovarian cancer: course over time and associated factors

Majke H.D. van Bommel[1], Miranda P. Steenbeek[1], Joanna IntHout[2], Rosella P.M.G. Hermens[3], Nicoline Hoogerbrugge[4], Marline G. Harmsen[1], Helena C. van Doorn[5], Marian J.E. Mourits[6], Marc van Beurden[7], Ronald P. Zweemer[8], Katja N. Gaarenstroom[9], Brigitte F.M. Slangen[10], Monique M.A. Brood-van Zanten[7,11], M. Caroline Vos[12], Jurgen M. Piek[13], Luc R.C.W. van Lonkhuijzen[11], Mirjam J.A. Apperloo[14], Sjors F.P.J. Coppus[15], Judith B. Prins[16], José A.E. Custers[16], Joanne A. de Hullu[1]

Corresponding author

Name: Majke van Bommel

Affiliation: Radboud university medical center, Radboud Institute for Health Sciences, Department of Obstetrics and Gynaecology, Nijmegen, The Netherlands.

E-mail address: majke.vanbommel@radboudumc.nl

Online Resource 3. *Variables associated with pre-surgical cancer worry in the three groups based on a validated cut-off score*

		**Persistent low (n=173)**	**Fluctuating (n=209)**	**Persistent high (n=106)**	**Total group (n=577)**	***p*-value**
BRCA-PV type, % *BRCA1* / % *BRCA2* [a]		49.1 / 50.9	51.7 / 48.3	51.9 / 48.1	51.5 / 48.5	.858
Years since *BRCA* diagnosis, mean (SD) [b]		5.4 (4.7)	4.7 (4.6)	4.6 (4.5)	5.0 (4.6)	.208
Personal history of breast cancer, %[a]		9.2	11.0	31.1	14.3	<.001
Emotional instability, % [a]		11.0	19.6	22.6	17.2	.021
Previous risk reducing mastectomy, % [a]		42.2	39.2	34.0	38.6	.392
Working a job, % [a]		87.9	85.2	78.3	83.5	.108
Breast cancer risk perception, mean (SD) [b]	*BRCA1*	40.4 (35.5)	40.8 (34.3)	38.0 (34.2)	39.7 (34.4)	.891
	BRCA2	43.5 (31.6)	50.6 (29.6)	53.9 (30.1)	49.7 (30.7)	.096
Ovarian cancer risk perception, mean (SD) [b]	*BRCA1*	43.8 (18.4)	45.4 (17.3)	48.2 (22.6)	45.7 (18.9)	.603
	BRCA2	22.8 (16.4)	23.7 (18.1)	33.1 (26.0)	25.2 (19.2)	.099

BRCA-PV, *BRCA* pathogenic variant; NS, not significant; SD, standard deviation

[a] analysed using Chi-square test; [b] analysed using Kruskal-Wallis test
