# Supplementary material for: Cancer worry among BRCA1/2 pathogenic variant carriers choosing surgery to prevent tubal/ovarian cancer: course over time and associated factors
Source: Support Care Cancer. 2022 Jan 8;30(4):3409–18. doi: 10.1007/s00520-021-06726-4 (PMC8857097; doi:10.1007/s00520-021-06726-4)
Supplement: Supplementary file 4 — Supplementary file4 (PDF 122 KB) [file 520_2021_6726_MOESM4_ESM.pdf]

Cancer Worry among *BRCA1/2* pathogenic variant carriers choosing surgery to prevent tubal/ovarian cancer: course over time and associated factors

Majke H.D. van Bommel[1], Miranda P. Steenbeek[1], Joanna IntHout[2], Rosella P.M.G. Hermens[3], Nicoline Hoogerbrugge[4], Marline G. Harmsen[1], Helena C. van Doorn[5], Marian J.E. Mourits[6], Marc van Beurden[7], Ronald P. Zweemer[8], Katja N. Gaarenstroom[9], Brigitte F.M. Slangen[10], Monique M.A. Brood-van Zanten[7,11], M. Caroline Vos[12], Jurgen M. Piek[13], Luc R.C.W. van Lonkhuijzen[11], Mirjam J.A. Apperloo[14], Sjors F.P.J. Coppus[15], Judith B. Prins[16], José A.E. Custers[16], Joanne A. de Hullu[1]

Corresponding author

Name: Majke van Bommel

Affiliation: Radboud university medical center, Radboud Institute for Health Sciences, Department of Obstetrics and Gynaecology, Nijmegen, The Netherlands.

E-mail address: majke.vanbommel@radboudumc.nl

Online Resource 4. *Extension of Table 3, characteristics that were evaluated whether or not they were associated with membership in a class with similar patterns of cancer worry over time; low declining class versus high stable and high declining class*

		Multinomial logistic regression		
	Class	OR	95%CI	*p*-value
BRCA2-PV type	Low declining	1		
	High stable	1.822	0.865; 3.839	.114
	High declining	0.849	0.591; 1.219	.374
Married/relationship	Low declining	1		
	High stable	1.223	0.346; 4.324	.754
	High declining	1.729	0.951; 3.143	.073
Risk-reducing mastectomy	Low declining	1		
	High stable	1.043	0.500; 2.177	.910
	High declining	0.697	0.479; 1.013	.059
Choice for novel strategy	Low declining	1		
	High stable	1.423	0.595; 3.406	.428
	High declining	0.958	0.641; 1.431	.834
Previous cancer other than breast cancer	Low declining	1		
	High stable	.[a]	.	.
	High declining	2.277	0.377; 13.754	.370
Use of anti-depressants	Low declining	1		
	High stable	.[a]	.	.
	High declining	1.773	0.864; 3.639	.119
First degree family history of breast cancer	Low declining	1		
	High stable	0.666	0.308; 1.439	.301
	High declining	1.287	0.896; 1.849	.172
First degree family history of ovarian cancer	Low declining	1		
	High stable	1.037	0.379; 2.839	.944
	High declining	0.721	0.421; 1.233	.232
Offspring	Low declining	1		
	High stable	1.709	0.389; 7.511	.478
	High declining	0.802	0.457; 1.405	.440
Age, years	Low declining	1		
	High stable	0.910	0.823; 1.005	.063

	High declining	0.992	0.942; 1.044	.751
Breast cancer risk perception (per point of increased perceived risk)	Low declining	1		
	High stable	1.011	0.999; 1.023	.063
	High declining	1.005	1.000; 1.011	.074

OR, Odds ratio; CI, confidence interval

[a] cannot be calculated because n=0
